# Exploring Risk and Resilient Profiles for Functional Impairment and Baseline Predictors in a 2-Year Follow-Up First-Episode Psychosis Cohort Using Latent Class Growth Analysis

**DOI:** 10.3390/jcm10010073

**Published:** 2020-12-28

**Authors:** Estela Salagre, Iria Grande, Brisa Solé, Gisela Mezquida, Manuel J. Cuesta, Covadonga M. Díaz-Caneja, Silvia Amoretti, Antonio Lobo, Ana González-Pinto, Carmen Moreno, Laura Pina-Camacho, Iluminada Corripio, Immaculada Baeza, Daniel Bergé, Norma Verdolini, André F. Carvalho, Eduard Vieta, Miquel Bernardo, PEPs Group

**Affiliations:** 1 Bipolar and Depressive Disorders Unit, Hospital Clinic, Biomedical Research Networking Center for Mental Health Network (CIBERSAM), August Pi I Sunyer Biomedical Research Institute (IDIBAPS), University of Barcelona, 08036 Barcelona, Spain; esalagre@clinic.cat (E.S.); bsole@clinic.cat (B.S.); nverdolini@clinic.cat (N.V.); 2 Barcelona Clinic Schizophrenia Unit, Neuroscience Institute, Hospital Clinic of Barcelona, Biomedical Research Networking Center for Mental Health Network (CIBERSAM), Department of Medicine, Institut de Neurociències, August Pi I Sunyer Biomedical Research Institute (IDIBAPS), Universitat de Barcelona, 08036 Barcelona, Spain; mezquida@clinic.cat (G.M.); amoretti@clinic.cat (S.A.); bernardo@clinic.cat (M.B.); 3 Department of Psychiatry, Instituto de Investigaciones Sanitarias de Navarra (IdiSNa), Complejo Hospitalario de Navarra, 31008 Pamplona, Spain; mj.cuesta.zorita@navarra.es; 4 Department of Child and Adolescent Psychiatry, Institute of Psychiatry and Mental Health, Hospital General Universitario Gregorio Marañón, IiSGM, CIBERSAM, School of Medicine, Universidad Complutense, 28007 Madrid, Spain; covadonga.martinez@iisgm.com (C.M.D.-C.); cmoreno@hggm.es (C.M.); lpina@iisgm.com (L.P.-C.); 5 Department of Medicine and Psychiatry, Instituto de Investigación Sanitaria Aragón (IIS Aragón), Universidad de Zaragoza, 50009 Zaragoza, Spain; alobo@unizar.es; 6 Department of Psychiatry, Hospital Universitario de Alava, BIOARABA Health Research Institute, University of the Basque Country, 01009 Vitoria, Spain; anamaria.gonzalez-pintoarrillaga@osakidetza.eus; 7 Centro de Investigación Biomédica en Red de Salud Mental (CIBERSAM), 28029 Madrid, Spain; icorripio@santpau.cat; 8 Department of Psychiatry, Biomedical Research Institute Sant Pau (IIB-SANT PAU), Hospital Sant Pau, Universitat Autònoma de Barcelona (UAB), 08041 Barcelona, Spain; 9 Biomedical Research Networking Center for Mental Health Network (CIBERSAM), Child and Adolescent Psychiatry and Psychology Department, August Pi I Sunyer Biomedical Research Institute (IDIBAPS), Hospital Clínic of Barcelona, SGR-881, Universitat de Barcelona, 08036 Barcelona, Spain; ibaeza@clinic.cat; 10 Hospital del Mar Medical Research Institute, CIBERSAM, Autonomous University of Barcelona, 08003 Barcelona, Spain; DBerge@parcdesalutmar.cat; 11 Centre for Addiction and Mental Health, Department of Psychiatry, University of Toronto, Toronto, ON M6J 1H4, Canada; andrefc7@hotmail.com; 12 The IMPACT (Innovation in Mental and Physical Health and Clinical Treatment) Strategic Research Centre, School of Medicine, Barwon Health, Deakin University, Geelong, VIC 3220, Australia

**Keywords:** first-episode psychosis, functional outcomes, risk factors, early intervention, neurocognition, latent class analysis, precision medicine

## Abstract

Being able to predict functional outcomes after First-Episode Psychosis (FEP) is a major goal in psychiatry. Thus, we aimed to identify trajectories of psychosocial functioning in a FEP cohort followed-up for 2 years in order to find premorbid/baseline predictors for each trajectory. Additionally, we explored diagnosis distribution within the different trajectories. A total of 261 adults with FEP were included. Latent class growth analysis identified four distinct trajectories: Mild impairment-Improving trajectory (Mi-I) (38.31% of the sample), Moderate impairment-Stable trajectory (Mo-S) (18.39%), Severe impairment-Improving trajectory (Se-I) (12.26%), and Severe impairment-Stable trajectory (Se-S) (31.03%). Participants in the Mi-I trajectory were more likely to have higher parental socioeconomic status, less severe baseline depressive and negative symptoms, and better premorbid adjustment than individuals in the Se-S trajectory. Participants in the Se-I trajectory were more likely to have better baseline verbal learning and memory and better premorbid adjustment than those in the Se-S trajectory. Lower baseline positive symptoms predicted a Mo-S trajectory vs. Se-S trajectory. Diagnoses of Bipolar disorder and Other psychoses were more prevalent among individuals falling into Mi-I trajectory. Our findings suggest four distinct trajectories of psychosocial functioning after FEP. We also identified social, clinical, and cognitive factors associated with more resilient trajectories, thus providing insights for early interventions targeting psychosocial functioning.

## 1. Introduction

Psychosocial functioning refers to the ability to perform in daily living activities such as work, studies or recreational activities, and to establish satisfying interpersonal relationships with others [1]. In the last 50 years, psychiatry has progressively moved from a deficit-based care (which focuses on symptomatic remission), to a model oriented towards functional recovery, meaning that helping the patient to meet his/her personal goals has become as critical as achieving symptomatic remission [2,3]. In fact, it is increasingly accepted that functional outcomes are more meaningful when measuring treatment response than are scores on various scales rating only psychiatric symptoms [4]—and more aligned with what the patient ultimately expects from treatment [5]. Therefore, full functional remission is currently a preeminent goal in psychiatry.

Prior evidence suggests that achieving full functional recovery short after first-episode psychosis (FEP) is a stronger predictor of long-term full functional remission than symptomatic remission [6,7]. This evidence underscores the need to find early and modifiable factors associated with functional impairment already from early stages. Although multiple studies have investigated putative predictors of poor psychosocial functioning after FEP [8], most of them have approached this question using a dichotomous outcome, that is, presence vs. absence of functional impairment. The real picture seems far more complex, though, given the highly divergent outcomes in psychosocial functioning that individuals can experience after FEP, which encompass varying degrees of functional difficulties and different evolutions over time. Some patients will experience an early functional recovery, others might exhibit severe functional difficulties from illness onset and some subgroups might experience (persistent or transitory) mild to moderate functional impairment, which still have a negative impact on their daily life. Hence, the real challenge is to predict early in the course of the disease which individual will fall into each of these trajectories in order to be able to design earlier and more tailored treatments for social and personal recovery [2,9,10,11].

Statistical methods like latent class growth analysis (LCGA) can help to provide a more accurate picture of the heterogeneous course in psychosocial functioning that can be observed following FEP, as it allows considering different outcomes of the same characteristic simultaneously [12,13]. To our knowledge, only few studies so far have applied these statistical techniques to assess functional outcomes in FEP samples [14,15,16], and none of them has considered simultaneously sociodemographic variables, clinical features and an extensive set of cognitive domains, all of them previously related to poor functional outcomes [17]. Therefore, our main aim was to identify different trajectories of functional impairment in the 24-month follow-up of a FEP cohort and to assess putative predictors of these diverse trajectories, with a special focus on resilient trajectories. As a secondary objective, we aimed to explore diagnoses distribution within the different trajectories. 

## 2. Experimental Section

### 2.1. Participants

The current study is based on data from the project ‘Phenotype–genotype and environmental interaction. Application of a predictive model in first psychotic episodes’ (PEPs study), a multicenter, longitudinal, naturalistic follow-up study [18]. A total of 16 centers throughout Spain participated in this study; fourteen of them were members of the Biomedical Research Networking Center for Mental Health (CIBERSAM) [19] and two were collaborator centers [18]. The study was conducted in accordance with the ethical principles of the Declaration of Helsinki. It was approved by the ethics committees at each participating center (project identification code: 2008/4232). All participants or their legal guardians signed an informed consent after providing them a full explanation of the study’s procedures.

The detailed protocol of the PEPs study was published elsewhere [18,20]. Briefly, a total of 335 subjects with FEP were recruited by all the participating centers, from April 2009 to April 2012. Individuals were included in the PEPs study if they were between 7 and 35 years old, presented first lifetime psychotic symptoms for at least one week in the last 12 months, were fluent in Spanish language, and were willing to sign the informed consent. Intellectual disability according to the Diagnostic and Statistical Manual of mental disorders, 4th edition (DSM-IV) criteria [21], history of head trauma with loss of consciousness, and presence of an organic disease with mental repercussions constituted exclusion criteria. Patients had been under antipsychotic treatment for less than 12 months at study entry. Follow-up assessments were conducted at 2 months, 6 months, 12 months and 24 months following inclusion.

### 2.2. Assessment

#### 2.2.1. Baseline Sociodemographic Data

Sociodemographic data were collected from all participants at baseline, including sex, age, ethnicity, educational level, marital status, current living situation, occupation, and parental socioeconomic status (SES). Parental SES was determined using the Hollingshead Two-Factor Index of Social Position [22]. Personal and family history of somatic and psychiatric disorders was also compiled. History of drug misuse was evaluated using the adapted version of a Multidimensional Assessment Instrument for Drug and Alcohol Dependence scale [23]. The Family Environment Scale (FES), a self-report instrument, was used to assess the patients’ perception of the social climate within their families [24,25].

#### 2.2.2. Baseline Clinical and Functional Assessment

For all subjects in the study, diagnosis was established by experienced mental health professionals using the Structured Clinical Interview for DSM-IV Axis I Disorders (SCID-I) [21,26]. Psychopathology was evaluated using the Spanish validated versions of the Positive and Negative Syndrome Scale (PANSS) [27,28], the Young Mania Rating Scale (YMRS) [29,30], and the Montgomery–Åsberg Depression Rating Scale (MADRS) [31,32]. Premorbid adjustment was estimated by means of the retrospective Premorbid Adjustment Scale (PAS) [33]. The Functional Assessment Short Test (FAST) [1,34] was used to determine psychosocial functioning. It comprises 24 items, which evaluate six specific functioning domains: autonomy, occupational functioning, cognitive functioning, financial issues, interpersonal relationships, and leisure time. This scale seeks to identify changes or difficulties in functionality attributable to the illness. The FAST scores range from 0 to 72. According to the cut-off classification as proposed by Bonnín et al. [35], FAST scores > 40 are indicative of severe functional impairment, FAST score between 21 and 40 indicate moderate functional impairment, FAST scores between 12–20 indicate mild impairment, and ≤11 points in the FAST reflect no functional impairment. This scale has shown to be sensitive to change and has been validated for FEP [36]. In all the aforementioned scales, higher scores are indicative of greater clinical severity or functional impairment. History of traumatic life events was assessed through the Spanish version of the Trauma Questionnaire (TQ) [37,38]. Duration of untreated psychosis (DUP), defined as the number of days elapsed between the onset of positive psychotic symptoms and the initiation of the first appropriate treatment for psychosis, was also registered. It was estimated using the Symptom Onset in Schizophrenia (SOS) inventory [39].

#### 2.2.3. 2-Month Follow-Up Neuropsychological Assessment

Participants were likewise evaluated using a comprehensive neuropsychological battery encompassing most of the cognitive domains proposed by the National Institute of Mental Health MATRICS consensus [40]. The evaluation was performed by trained neuropsychologists in the first two months after the inclusion of the participant in the study to avoid the interference of acute psychopathological manifestations on neurocognitive assessments. The neuropsychological assessment comprised the following cognitive domains: (1) estimated Intelligence Quotient (IQ) (calculated based on the performance on the Vocabulary subtest from the Wechsler Adult Intelligence Scale (WAIS-III) [41]); (2) executive function (Stroop Color-Word Interference Test [42], Wisconsin Card Sorting Test (WCST) [43] and Trail Making Test (TMT), form B [44]); (3) attention (Continuous Performance Test-II (CPT-II) [45]); (4) processing speed (TMT, form A [46] and categorical (Animal Naming) and phonemic (F-A-S) components of the Controlled Oral Word Association Test (COWAT) [47]); (5) verbal memory (Spanish version of the California Verbal Learning Test, the *Test de Aprendizaje Verbal España-Complutense* (TAVEC) [48]); (6) working memory (Digit span and Letter-Number sequencing subtests of WAIS-III [41]); and (7) social cognition (Mayer–Salovey–Caruso Emotional Intelligence Test (MSCEIT) [49,50]). The neuropsychological battery is described in further detail in the PEPsCog study [51].

### 2.3. Statistical Analysis

#### 2.3.1. Identification of Functional Trajectories: Latent Class Growth Analysis

LCGA was used to identify distinct functioning trajectories over the 24-month follow-up. In the current analysis, individual class membership was assigned on the basis of FAST total scores measured at five time points over the two-year follow-up period, namely at baseline, 2-, 6-, 12-, and 24-month follow-up. We only included in the analysis individuals over 18 years old, as the FAST scale has only been validated in adult samples, and with information on the FAST scale in at least two follow-up assessments. This left a sample of 275 adult participants.

Each model was rerun 100 times using different start values to avoid converging to local maxima [52]. To accommodate expected fluctuations over time, we estimated linear and quadratic terms. In order to determine the optimal number of trajectory classes, models with increasing number of latent classes (from 1- to 4-class models) were fitted to the data and the best-fitting model was selected according to the following goodness-of-fit indices: Akaike’s Information Criterion (AIC), Bayesian Information Criterion (BIC), samples-size-adjusted BIC (aBIC), and entropy. Lower values of AIC, BIC, and aBIC suggest a more parsimonious model, while higher entropy also indicates better model fit. Entropy ranges from 0 to 1 and is a summary indicator of the accuracy with which models classify individuals into their most likely class. Entropy with values approaching 1 indicate clear delineation of classes [53]. Interpretability and parsimony of the model were also taken into consideration in the final selection of the model. LCGA analyses were performed on R version 3.6.3, using the *‘lcmm’* package ([54]; https://cran.r-project.org/web/packages/lcmm/index.html).

#### 2.3.2. Identification of Baseline Predictors of Functional Trajectory Membership

To identify putative baseline predictors of trajectory membership, the estimated latent classes (i.e., the estimated trajectory group) derived from LCGA were imported to SPSS, version 23 (SPSS Inc., Chicago, IL, USA), for a three-step analysis: 

First, we created seven cognitive composites to be used as putative baseline predictors using data from the two-month follow-up neurocognitive assessment. To do so, patients’ raw scores on each neuropsychological task were standardized to z-scores based on the performance of the whole sample. The selection of the tasks within each cognitive domain was based on previous works from the PEPs group [51,55,56]. Afterwards, z-scores of different tests were summed and averaged to create the following seven cognitive composites: (1) the processing speed composite, based on the word–color task from the Stroop Test and the TMT-A; (2) the working memory composite, which included the Letter-Number Sequencing and the Digit-Span WAIS-III subtests; (3) the verbal learning and memory index, which was composed of the total trials 1–5 list A, short free recall, short cued recall, delayed free recall, delayed cued recall, and recognition scores of the TAVEC; (4) the executive function composite, calculated based on the number of categories and perseverative errors of the WCST, the Stroop Interference Test, and the TMT-B; (5) the attention composite score, which was based on several measures of the CPT-II, such as commission and reaction time; (6) the verbal fluency composite which was composed of the Category Fluency (Animal Naming) and the F-A-S Test of the COWAT; and (7) the social cognition composite, which included the Emotional Management of the MSCEIT. Whenever extreme scores in the performance of the aforementioned test were detected (i.e., more than four standard deviations (SD) above or below the mean), the scores were truncated to z = +/− 4. Since higher scores in CPT-II, WCST perseverative errors, and TMT-A and -B indicate poorer performance, z-scores obtained from measures of these tests were reversed before constructing the corresponding composite scores.

Second, candidate predictors (i.e., baseline sociodemographic and clinical variables as well as the created cognitive composites) were compared between trajectory classes using Kruskal-Wallis and chi-square tests, as appropriate. The Kruskal–Wallis test was selected for continuous variables since they did not follow a normal distribution, as assessed visually and by the Kolmogorov–Smirnov test. When applicable, post-hoc comparison analyses with Bonferroni correction for multiple comparisons were performed to further clarify the presence of significant differences between trajectory classes.

Third, those variables found to be statistically significant in the post-hoc analysis in at least two pair-wise comparisons were then entered into a multinomial regression model to determine which candidate factors independently predicted trajectory membership, adjusting for age and sex. For the PANSS scale, only the PANSS positive and negative subscales were entered as independent variables to avoid multicollinearity. Significant putative predictors for the multivariable model were identified using a stepwise backwards elimination process [57], with sex and age entered as fixed factors. The identified latent classes were used as the dependent variable. Since we were interested in exploring predictors of resilient trajectories, we selected the most impaired group as the reference category.

#### 2.3.3. Diagnosis Distribution within the Identified Functional Trajectories

Lastly, to explore whether diagnosis distribution differed within each functional trajectory and how it changed over time, we compared using chi-square tests the proportion of individuals with a diagnosis of Schizophrenia, Bipolar disorder, Schizoaffective disorder, and Other psychoses (including psychotic disorder not-otherwise specified, brief psychotic disorder, schizophreniform psychosis, delusional disorder, substance-induced psychosis) in each of the predicted functional trajectories at baseline, 1-year and 2-year follow-up.

The level of statistical significance for all analyses was set at *p* < 0.05.

## 3. Results

### 3.1. Sample Characteristics and Attrition Analysis

The final sample included 261 participants. A total of 14 individuals were not considered for the analyses since information on their FAST scores was only available at one time point. Therefore, they were treated as drop-outs. The baseline characteristics of the final sample are presented in Table 1. A comparison between drop-outs and non-drop-outs at baseline, 12-month, and 24-month follow-up can be found in the Supplementary Table S1. The median age of the final sample was 25.05 years old (Interquartile Range: 9) and 33% of the participants were female. Among those subjects that dropped out from the study, there was a lower proportion of Caucasian participants and of participants with a family history of psychiatric disorders. Subjects that dropped out from the study reported more frequently substance misuse at baseline too.

### 3.2. Latent Classes of Functional Trajectories

After examining fit indices, entropy, parsimony, and interpretability of the model, the 4-class model including the quadratic term was selected as optimal for our data (Table 2). Entropy was acceptable (0.76) for the 4-class model as well as post mean class probabilities (0.81 for Class 1, 0.92 for Class 2, 0.82 for Class 3, and 0.84 for Class 4). This suggests that with the 4-class model individuals were likely to be correctly assigned to their respective latent class.

**Figure 1:**
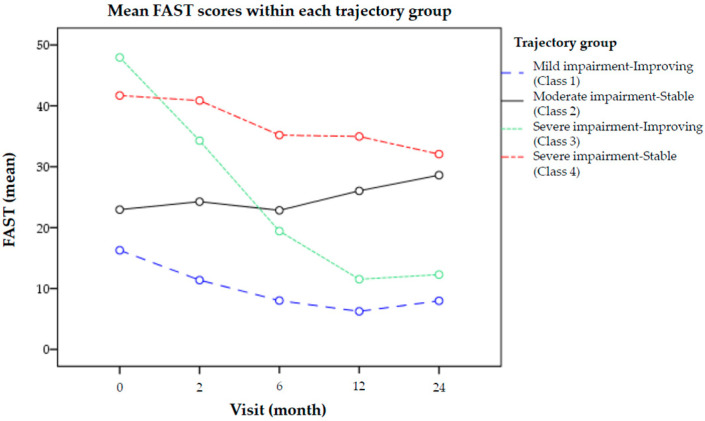
Evolution of mean FAST scores within each of the functional trajectory groups derived from the latent class growth analysis. Higher scores in the FAST are indicative of greater functional impairment.

The mean FAST scores at each assessment point of individuals grouped according to their predicted trajectory are presented in Figure 1. One group showed mild impairment at baseline and no impairment by the end of the follow-up, and was referred to as *Mild impairment-Improving trajectory* (Class 1; *n* = 100 (38.31%)). Another group, denominated as *Moderate impairment-Stable trajectory* (Class 2; *n* = 48 (18.39%)) exhibited moderate functional impairment at baseline and throughout the follow-up. A third group presented with severe functional impairment that improved along the follow-up. It was referred as *Severe impairment-Improving trajectory* (Class 3; *n* = 32 (12.26%)). The last group, termed as *Severe impairment-Stable trajectory*, displayed severe-moderate functional impairment throughout the follow-up (Class 4; *n* = 81 (31.03%)). Thus, 50.57% of the sample showed a trajectory characterized by a functional improvement/recovery (“*Improving trajectories*”), while 49.42% exhibited persistent functional impairment during follow-up (“*Stable trajectories*”).

### 3.3. Baseline Predictors of Trajectory Membership

The comparison between the four psychosocial functioning trajectories on sociodemographic, clinical, and neuropsychological variables is presented in Table 3. The baseline variables found to be statistically different between groups in at least two pairwise comparisons were: parental SES, alcohol use, PANSS positive, PANSS negative, PANSS general, PANSS total, Young total, MADRS total, PAS total, verbal learning, and memory and working memory. As previously stated, for the PANSS scale, only the PANSS positive and negative subscales were entered as independent variables in the multinomial regression model.

Multinomial regression analysis (final model: R^2^ Nagelkerke 53%, X^2^ = 140.26; df = 24; *p* < 0.001) indicated that parental SES, total baseline scores in PANSS positive subscale, PANSS negative subscale, MADRS, and PAS, as well as verbal learning and memory contributed to differentiate among the four functional trajectories (Table 4). Specifically, subjects falling into the *Mild impairment-Improving* group were more likely to have a medium-high parental SES (OR: 4.14, 95% CI 1.65–10.42), lower severity of baseline negative symptoms (OR: 0.89, 95% CI 0.83–0.96) and of depressive symptoms (OR: 0.94, 95% CI 0.89–0.99), and better premorbid adjustment (OR: 0.96, 95% CI 0.94–0.98). On the other hand, compared to individuals in the *Severe impairment-Stable* trajectory, better premorbid adjustment (OR: 0.96, 95% CI 0.93–0.99) and higher scores in the verbal learning and memory domain (OR: 3.09, 95% CI 1.36–7.03) increased the probability of belonging to the *Severe impairment-Improving trajectory* group. Finally, individuals falling in the *Moderate impairment-Stable* trajectory were more likely to score lower in the PANSS positive subscale (OR: 0.93, 95% CI 0.87–0.99) at baseline than the *Severe impairment-Stable* group.

### 3.4. Exploring Diagnoses Distribution among Functional Trajectories throughout the Follow-Up

**Figure 2:**
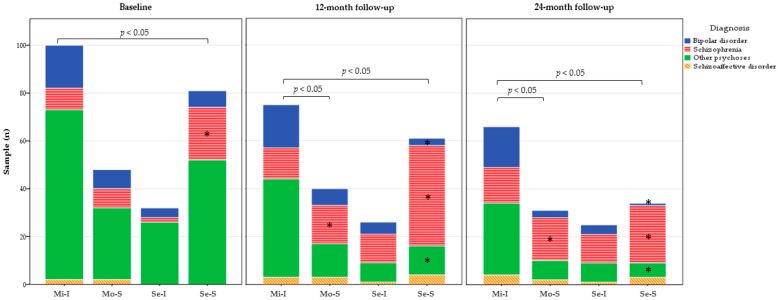
Diagnoses distribution within each of the identified functional trajectories.

The diagnoses distribution within each functional trajectory at baseline, one-year follow-up and two-year follow-up is depicted in Figure 2. Diagnosis distribution significantly differed between trajectory groups at baseline (*n* = 261; X^2^ = 19.9; *p* = 0.02), 1-year follow-up (*n* = 202; X^2^ = 42.6; *p* < 0.001) and at 2-year follow-up (*n* = 156; X^2^ = 28.5; *p* = 0.001). A higher proportion of patients with a diagnosis of Schizophrenia was found among individuals falling into the *Severe impairment-Stable* and *Moderate impairment-Stable* trajectories compared to the *Mild impairment-Improving trajectory*. On the other hand, the diagnoses of Bipolar disorder and Other psychosis were more frequent among individuals falling into the *Mild impairment-Improving trajectory* compared to the *Severe impairment-Stable* trajectory. Abbreviations: Mi-I: Mild impairment-Improving; Mo-S: Moderate impairment-Stable; Se-I: Severe impairment-Improving; Se-S: Severe impairment-Stable.

Figure 2 represents, for each of the functional trajectories derived from the Latent class growth analysis, the number of individuals with a diagnosis of Bipolar disorder, Schizophrenia, Schizoaffective disorder, or Other psychoses at baseline, 12-month and 24-month follow-up. Other psychoses include psychotic disorder not-otherwise specified, brief psychotic disorder, schizophreniform psychosis, delusional disorder, and substance-induced psychosis. (*) symbol indicates which diagnostic categories within the *Severe impairment-Stable* trajectory and the *Moderate impairment-Stable* trajectory show a significantly different proportion of individuals compared to the *Mild impairment-Improving* trajectory group. Abbreviations: Mi-I: Mild impairment-Improving; Mo-S: Moderate impairment-Stable; Se-I: Severe impairment-Improving; Se-S: Severe impairment-Stable

### 3.5. Post-Hoc Mediation Analysis

Given that previous works on FEP samples have suggested that premorbid adjustment may influence psychosocial functioning through verbal memory and negative symptoms [59], we decided to test how the identified predictors interact to impact functioning in our sample. For that, we examined mediation using a regression-based bootstrapping approach [60]. Analyses were performed with PROCESS [61], with age and sex introduced as covariables (see Appendix A for a more detailed explanation). The model used to explore mediation between predictors of the *Severe impairment-improving trajectory* vs. *Severe impairment-Stable trajectory* indicated that better premorbid adjustment positively impacts verbal learning and memory, which in turn increases the probability of belonging to the *Severe impairment-improving* trajectory (indirect effect = −0.011; 95% CI, −0.030 to −0.001). However, our results indicate complementary partial mediation since both direct and indirect effects were significant and pointed in the same direction [62]. Regarding mediation between predictors of *Mild impairment-Improving* vs. *Severe impairment-Stable* trajectories, we could establish that parental SES partially mediates its effects through premorbid adjustment and through baseline negative symptoms (indirect effect = −0.249; 95% CI, −0.551 to −0.086).

## 4. Discussion

In this study, we used LCGA to investigate trajectories of psychosocial functioning following FEP. In line with previous studies using the same approach [14,15,16], our results indicate a heterogeneous pattern of psychosocial functioning in the first years after FEP. Specifically, we found four distinct functional trajectories. The largest number of subjects in our sample showed mild functional impairment at baseline and experienced functional recovery short after FEP. The second largest group experienced severe functional impairment at baseline which persisted, although more moderately, throughout the study period. A third group displayed a moderate and persistent functional impairment throughout the 24-month follow-up. Finally, a minority of patients exhibited severe functional impairment at baseline, which subsequently improved almost to the point of no functional impairment by the end of the follow-up. Importantly, around 50% of the sample exhibited a marked functional improvement by the end of follow-up. Baseline factors associated with functional improvement were parental medium-high SES, less severe negative, and depressive symptoms (for individuals in the *Mild impairment-Improving* trajectory), better scores in the verbal learning and memory domain (for individuals in the *Severe impairment-Improving* trajectory) and better premorbid adjustment (for both the *Mild impairment-Improving* and *Severe impairment-Improving* trajectory groups). Less severe positive symptoms at baseline predicted a *Moderate impairment-Stable* trajectory vs. a *Severe impairment-Stable* trajectory. These results are in agreement with previous studies performed in FEP and chronic psychiatric samples, where parental SES [17,63], negative [14,64,65] and depressive symptoms [66,67], verbal memory [64], and premorbid adjustment [14,68] were predictors of functional outcomes. To our knowledge, however, this is the first study to simultaneously analyze such a large panel of potential predictors of mid-term psychosocial functioning trajectories identified using an LCGA approach, which included sociodemographic, clinical, and neurocognitive variables, and to further examine the interaction between the identified predictors.

Regarding diagnosis distribution among classes, our findings are in keeping with previous research [20,69]. All diagnoses were represented in the four trajectories, yet the proportion of patients with a diagnosis of Schizophrenia was higher among individuals showing persistent functional difficulties, whereas a higher proportion of patients with Bipolar disorder or Other psychoses fell into the group showing the most favorable functional trajectory. Despite these results need to be interpreted with caution due participants drop-out during follow-up, we found the same pattern at 12-month and 24-month follow-up.

In our study, medium-high parental SES appeared as one of the main predictors of the trajectory characterized by mild functional impairment at first assessment followed by an early functional recovery. The association between higher parental SES and better functional outcomes is probably a complex one. Our mediation analysis, indeed, suggests that parental SES partially mediate its influence on functionality through premorbid adjustment and negative symptoms. However, other factors not included in the mediation analysis also seem to play a role. For instance, families with a higher SES might provide more cognitive stimulation to their offspring [70], for example, involving them in more intellectual, artistic, or cultural leisure activities, hence enhancing their cognitive reserve, which has been associated with better functional outcomes [56,71,72]. In fact, we found that subjects within the *Mild impairment-Improving* trajectory reported to be involved in more social and recreational activities than the *Severe impairment-Improving* trajectory group, as reflected by higher scores in the Active-recreational orientation subscale of the FES. These families may likewise have more resources to identify the first psychotic symptoms and enable an earlier engagement with mental health services [73]. It could also translate more family support or means to provide better care in the post-FEP period [74]. In any case, our results emphasize the need for social interventions to promote and educate on mental health and facilitate the access to mental health services in the pre- and post-FEP period [75,76], as it has been done in Australia through the headspace initiative (https://www.headspace.org.au).

Several studies have consistently reported a relationship between verbal learning and memory and functional outcomes, both in affective and non-affective samples [51,67,77,78,79]. For instance, more preserved verbal learning before enrolling to functional remediation, a psychological therapy specifically targeting functional impairments, is associated with better long-term functional outcomes after this therapy [80]. Negative symptoms are also well-known predictors of poor functional outcomes [81,82,83] and the interrelationship between negative symptoms and cognition as predictors of functionality has been a matter of intense debate and study in prior works [84,85]. In the study by Milev et al. [64], performed in a sample of 99 subjects followed for seven years after FEP, verbal memory appeared as a strong predictor of global functioning in univariate logistic analysis. However, when the effect of verbal memory was examined together with negative symptoms in a multivariate multinomial logistic regression, negative symptoms took precedence over verbal memory as a predictor of global functioning, since the latter was no longer significant. In their three years follow-up study, Simons et al. [86] likewise found that the association between the performance in most cognitive domains, including verbal memory, and social functioning in the long-term was fully mediated by negative symptoms. Finally, Jordan et al. [59] showed that verbal memory predicted length of negative symptoms remission in FEP patients, which in turn predicted better functional performance. According to this evidence, negative symptoms might play a more predominant role predicting functional outcomes than verbal memory. That might explain why, when comparing those groups exhibiting significantly different severity of negative symptoms at baseline (i.e., *Severe impairment-Stable* vs. *Mild impairment-Improving*), negative symptoms but not verbal memory appeared as a predictor of poorer functional trajectory. In contrast, when comparing groups with similar negative symptoms at baseline (i.e., *Severe impairment-Stable* vs. *Severe impairment-Improving trajectory*), more preserved verbal memory arose as a significant predictor of better functional recovery. Consequently, our findings confirm the importance of negative symptoms as a treatment target for functional recovery and suggest that assessing performance in verbal learning and memory might be especially useful as a differential factor of future functional outcome in FEP subjects presenting with severe functional impairment and similar negative symptoms. On the contrary, for those subjects showing mild negative symptoms at baseline, assessing verbal memory and learning might not provide additional information on their functional prognosis.

Better premorbid adjustment also appeared as a predictor of a more favorable functional trajectory in our analysis, in keeping with prior evidence [81,87]. As suggested by Hodgekins et al. [14], the persistence in functional impairment after FEP in those subjects with poorer premorbid adjustment might just reflect a functional disability that was already present before the onset of the full-blown psychotic episode, then rendering it difficult for these patients to achieve a functional remission—hence, the importance of intervening early in the course of the disease with specific interventions designed to improve functionality [75,88,89]. Considering that the effects of premorbid adjustment on psychosocial functioning might be partially mediated by verbal learning and memory, as further supported by Jordan et al. [59], those individuals at high-risk for affective and non-affective psychosis who exhibit poor social adjusted (and especially those with low parental SES) might benefit from an adapted version of functional remediation, which improves functionality but also enhances verbal memory [90,91]. Randomized clinical trials in early-stage samples will be needed to test the real benefit of early functional remediation interventions (ideally adapted to high-risk samples) in long-term psychosocial outcomes. To date, evidence coming from randomized clinical trials is only available on the effect of cognitive remediation in individuals at ultra-high risk for psychosis, which points to a positive impact on cognitive measures, including verbal memory, but less clear effects on psychosocial functioning [92].

Finally, our results indicate that less severe depressive symptoms at baseline are associated with a *Mild impairment-Improving* trajectory. Persistent depressive symptoms have been shown to worsen functional prognosis after FEP [93,94]; however, in our study, we were evaluating the putative predictive role of baseline depressive symptoms and therefore it can be that our findings just reflect a less severe clinical presentation in the *Mild impairment-Improving* trajectory compared to the *Severe impairment-Stable* trajectory. Additionally, the *Severe impairment-Stable* trajectory was characterized by more severe negative symptoms, and we cannot rule out some overlap between scores in the MADRS and the PANSS negative subscale [95]. A similar explanation can be applied to our findings of lower scores at baseline in the PANSS positive subscale being predictors of a *Moderate impairment-Stable* trajectory compared to the *Severe impairment-Stable* trajectory. They may reflect that the differences in functionality observed between the two groups in the first assessment are driven by more severe psychotic symptoms at baseline. 

Future works with greater sample size, including variables not available in this study (such as cognitive reserve scores or biological markers) and taking into account longitudinal factors that can also influence functioning (such as persistent substance abuse or therapeutic non-compliance) would be needed to confirm and refine our findings. Furthermore, our findings that all diagnoses are represented in all trajectories support the idea that there are transdiagnostic subgroups that are alike in clinical presentation and outcomes. According to previous research [96], these subsets of patients might represent specific biotypes that are not governed by classical diagnostic criteria. Therefore, future studies that analyze whether patients falling in resilient vs. persistent functional trajectories are characterized by a differential set of biomarkers would be interesting to develop precise models of risk stratification of functional impairment. For now, our results already suggest that more preserved verbal learning and memory could be used as a marker of functional resilience in those FEP patients with a more severe clinical and functional presentation.

## 5. Limitations

The current study presents several limitations to be noted. Firstly, as a sub-analysis of a prior study not primarily designed for the purpose of the present work, sample size might be too small and follow-up too short to capture all the potential trajectories for psychosocial functioning. Secondly, trajectory “naming” is a subjective process; in our case, it was based on what we considered the most important information to be extracted from the observed trajectories. Some might not agree with the chosen labels for each trajectory. Nevertheless, we consider our approach to be pragmatic and clinically useful, as it delineates two subsets of patients: those at risk of sustained functional difficulties and those more resilient, that is, showing more improvement during follow-up. Thirdly, we focused on baseline predictors and did not take into account variables like treatment compliance or substance abuse during follow-up, which might also contribute to functional outcomes in the period after FEP. Fourthly, as the study design was constructed prior to 2009, specific scales for negative symptoms such as the Brief Negative Symptom Scale (BNSS) [97] or the Clinical Assessment Interview for Negative Symptoms (CAINS) [98] were not used. The same applies to cognitive reserve, with scales such as the CRASH not being available at that time [99]. Lastly, results regarding diagnosis distribution need to be interpreted with caution due to the small sample size in some of the diagnostic categories, which may render X^2^ results non-valid.

## 6. Conclusions

In our study, we identified four trajectories of psychosocial functioning following FEP, two of them indicative of a persistent functional impairment course and two describing a more resilient course. Additionally, our findings give some clues on putative factors that might mediate functional resilience, such as better socioeconomic status and premorbid adjustment, lesser negative symptoms, and more preserved verbal learning and memory. They also highlight that final functional outcomes are the result of the additive effects of a variety of factors. Hence, an integrative approach from very early stages is needed to target functional impairments, especially among those in a more vulnerable psychosocial situation.

## Figures and Tables

**Table 1:** Baseline characteristics of the final sample (*n* = 261).

Characteristics	Median (IQR)/*n* (%)
Age (Years)	25.05 (9)
Sex (Female)	87 (33.3)
Marital status (Single)	222 (85.1)
Ethnicity (Caucasian)	228 (87.4)
Parental socioeconomic status (Medium-high)	141 (54.6)
Living situation (Living independently)	55 (21.1)
Educational level (Higher education)	115 (44.2)
Occupational status (Active *)	136 (52.1)
Somatic comorbidity (Yes)	73 (28.0)
Family history of psychiatric disorder (Yes)	146 (55.9)

* Active includes workers and students.

**Table 2:** Goodness-of-fit statistics of latent class growth analysis with one-to-four class solutions of psychosocial functioning trajectories.

		Fit Statistics ^a^				% of the Sample in Each Class			
Number of Classes	Number of Parameters	AIC	BIC	aBIC	Entropy	Class 1	Class 2	Class 3	Class 4
1	4	9029.81	9044.07	9031.39	-	100	-	-	-
2	8	8675.19	8703.71	8678.35	0.82	54.02	45.98	-	-
3	12	8639.13	8681.91	8643.86	0.71	41.00	32.18	26.82	-
**4**	**16**	**8574.11**	**8631.14**	**8580.41**	**0.76**	**38.31**	**18.39**	**12.26**	**31.03**

Abbreviations: AIC: Akaike’s Information Criterion; BIC: Bayesian Information Criterion; aBIC: sample size–adjusted Bayesian Information Criterion. ^a^ Lower values (AIC, BIC, and aBIC) indicate a better model fit. Higher entropy indicates better model fit. Values of 0.4, 0.6, and 0.8 represent low, medium, and high entropy [58]. Bold is used here to indicate which model was selected.

**Table 3:** Comparison between groups derived from the identified functional trajectories.

	Mi-I (1) *n* = 100	Mo-S (2) *n* = 48	Se-I (3) *n* = 32	Se-S (4) *n* = 81	Kruskal–Wallis/X^2^	*p*-Value	Post-Hoc ^a^					
							1 vs. 2	1 vs. 3	1 vs. 4	2 vs. 3	2 vs. 4	3 vs. 4
***Sociodemographic characteristics***												
Age (years) ^b^	25.8 (10)	24.9 (8)	24.4 (8)	24.8 (8)	1.44	0.70						
Sex (Female) ^c^	29 (29.0)	18 (37.5)	10 (31.2)	30 (37.0)	1.78	0.62						
Ethnicity (Caucasian) ^c^	91 (91.0)	41 (85.4)	27 (84.4)	69 (85.2)	1.97	0.58						
Marital status (Single) ^c^	83 (83.0)	42 (87.5)	29 (90.6)	68 (83.9)	1.42	0.70						
Living situation (Living independent) ^c^	30 (30.0)	4 (8.3)	7 (21.9)	14 (17.3)	10.19	**0.02**	**<0.05**					
Educational level (Higher education) ^c^	55 (55.0)	20 (41.7)	16 (50.0)	24 (29.6)	12.71	**<0.01**			**<0.05**			
Occupational status (Active ^d^) ^c^	63 (63.0)	26 (54.2)	18 (56.2)	29 (35.8)	13.68	**<0.01**			**<0.05**			
Socioeconomic status (Medium-high) ^c^	72 (72.0)	22 (45.8)	18 (56.2)	29 (35.8)	24.06	**<0.001**	**<0.05**		**<0.05**			
Family history of psychiatric disorders (Yes) ^c^	53 (53.0)	33 (68.7)	17 (53.1)	43 (53.1)	3.92	0.27						
Previous psychiatric diagnoses (Yes) ^c^	24 (24.0)	13 (27.1)	8 (25.0)	29 (35.8)	4.65	0.59						
Substance use ^c^												
Tobacco	70 (70.0)	33 (68.7)	24 (75.0)	55 (67.9)	0.57	0.90						
Alcohol	60 (60.0)	25 (52.1)	24 (75.0)	32 (39.5)	14.05	**<0.01**			**<0.05**			**<0.05**
Cannabis	44 (44.0)	22 (45.8)	13 (40.6)	36 (44.4)	0.22	0.97						
Cocaine	9 (9.0)	8 (16.7)	6 (18.7)	12 (14.8)	3.04	0.39						
***Clinical measures***												
DUP (days) ^b^	85.0 (165)	133.0 (263)	110.0 (168)	162.0 (216)	14.36	**<0.01**			**<0.05**			
PANSS ^b^												
PANSS positive	14.0 (14)	16.0 (10)	23.5 (10)	21.0 (9)	32.14	**<0.001**		**<0.05**	**<0.05**	**<0.05**	**<0.05**	
PANSS negative	14.0 (11)	19.0 (13)	19.5 (11)	23.0 (9)	57.27	**<0.001**	**<0.05**	**<0.05**	**<0.05**		**<0.05**	
PANSS general	29.5 (19)	34.5 (17)	45.5 (19)	43.0 (14)	58.89	**<0.001**		**<0.05**	**<0.05**	**<0.05**	**<0.05**	
PANSS total	57.5 (39)	72.0 (28)	86.5 (29)	88.0 (24)	65.12	**<0.001**		**<0.05**	**<0.05**	**<0.05**	**<0.05**	
Young total ^b^	2.0 (14)	2.0 (14)	13.5 (19)	7.0 (18)	15.26	**<0.01**		**<0.05**		**<0.05**		
MADRS total ^b^	6.0 (10)	13.0 (12)	16.0 (17)	16.0 (12)	39.05	**<0.001**	**<0.05**	**<0.05**	**<0.05**			
PAS total ^b^	30.0 (24)	43.0 (27)	36.0 (31)	57.0 (33)	51.58	**<0.001**	**<0.05**		**<0.05**			**<0.05**
TQ ^b^	1.0 (2)	0.0 (1)	0.0 (2)	0.0 (2)	2.69	0.44						
FES ^b^												
Cohesion	52.0 (13)	52.0 (13)	52.0 (15)	47.0 (17)	5.30	0.15						
Expressiveness	53.0 (16)	50.0 (16)	53.0 (14)	47.0 (16)	4.14	0.25						
Conflict	49.0 (9)	49.0 (9)	49.0 (9)	49.0 (17)	7.19	0.07						
Independence	51.0 (11)	51.0 (11)	51.0 (14)	51.0 (17)	3.50	0.32						
Achievement-orientation	47.0 (10)	47.0 (10)	47.0 (15)	47.0 (16)	1.35	0.72						
Intellectual-cultural orientation	51.0 (23)	47.0 (14)	47.0 (14)	42.0 (19)	5.78	0.12						
Active-recreational orientation	53.0 (14)	48.0 (19)	48.0 (21)	44.0 (4)	19.69	<0.001			**<0.05**			
Moral-religious emphasis	44.0 (10)	49.0 (15)	44.0 (10)	44.0 (15)	3.34	0.34						
Organization	54.0 (10)	51.5 (19)	49.0 (20)	49.0 (15)	4.51	0.21						
Control	45.0 (14)	49.0 (14)	49.0 (14)	49.0 (14)	4.06	0.25						
***Cognitive measures***												
	*n* = 93	*n* = 44	*n* = 28	*n* = 76								
IQ ^b^	100 (20)	92.5 (24)	93.5 (19)	90.0 (20)	10.18	**0.02**			**<0.05**			
	*n* = 91	*n* = 43	*n* = 25	*n* = 67								
Verbal Fluency ^b^	0.20 (1.2)	−0.08 (1.4)	0.25 (1.1)	−0.50 (0.9)	20.69	**<0.001**			**<0.05**			
	*n* = 85	*n* = 36	*n* = 23	*n* = 53								
Attention ^b^	0.18 (0.4)	0.03 (0.7)	0.10 (0.6)	−0.11 (0.5)	13.54	**<0.01**			**<0.05**			
	*n* = 94	*n* = 43	*n* = 28	*n* = 73								
Working memory ^b^	0.14 (1.1)	−0.01 (1.3)	0.15 (0.9)	−0.17 (1.2)	14.10	**<0.01**			**<0.05**			**<0.05**
	*n* = 92	*n* = 40	*n* = 25	*n* = 67								
Verbal Learning and Memory ^b^	0.38 (1.2)	0.20 (1.3)	0.25 (0.9)	−0.43 (1.4)	24.18	**<0.001**			**<0.05**			**<0.05**
	*n* = 93	*n* = 41	*n* = 30	*n* = 70								
Processing Speed ^b^	0.32 (0.8)	0.14 (0.9)	−0.11 (1.4)	−0.16 (1.0)	14.78	**<0.01**			**<0.05**			
	*n* = 92	*n* = 40	*n* = 28	*n* = 58								
Executive function ^b^	0.29 (0.7)	−0.03 (0.8)	0.14 (0.8)	0.00 (1.0)	13.26	**<0.01**			**<0.05**			
	*n* = 89	*n* = 43	*n* = 26	*n* = 66								
Social cognition ^b^	−0.33 (1.3)	−0.01 (1.4)	0.04 (1.3)	−0.07 (1.4)	3.47	0.32						

^a^ Tukey or Z statistic, as appropriate. Significance values have been adjusted using the Bonferroni correction for multiple tests. Bold type indicates *p* < 0.05. ^b^ Values are indicated as median (Interquartile Range). ^c^ Values are indicated as *n* (%). ^d^ Active includes workers and students. Abbreviations: Mi-I: Mild impairment-Improving; Mo-S: Moderate impairment-Stable; Se-I: Severe impairment-Improving; Se-S: Severe impairment-Stable; DUP: Duration of Untreated Psychosis; PANSS: Positive and Negative Syndrome Scale; MADRS: Montgomery–Åsberg Depression Scale; YMRS: Young Rating Mania Scale; PAS: Premorbid Adjustment Scale; TQ: trauma questionnaire; FES: Family Environment Scale; IQ: Intelligence Quotient.

**Table 4:** Multinomial logistic regression ^a^: baseline predictors of functional trajectories.

	B	SE	Wald ^c^	Sig.	Adjusted Exp(B) ^b^	95% Interval Confidence Exp(B)	
						Lower Limit	Upper Limit
Mild impairment-improving trajectory *							
Intersection	4.95	1.62	9.35	**<0.01**			
Parental SES (medium-high)	1.42	0.47	9.14	**<0.01**	4.14	1.65	10.42
PANSS positive (baseline score)	−0.05	0.03	2.94	0.09	0.95	0.89	1.01
PANSS negative (baseline score)	−0.12	0.04	9.75	**<0.01**	0.89	0.83	0.96
MADRS total (baseline score)	−0.06	0.03	4.44	**0.03**	0.94	0.89	0.99
PAS total (baseline score)	−0.04	0.01	10.91	**<0.01**	0.96	0.94	0.98
Verbal learning & memory	0.39	0.28	1.89	0.17	1.47	0.85	2.55
Moderate impairment-stable trajectory *							
Intersection	3.58	1.65	4.71	**0.03**			
Parental SES (medium-high)	0.58	0.48	1.48	0.22	1.78	0.70	4.54
PANSS positive (baseline score)	−0.07	0.03	4.94	**0.03**	0.93	0.87	0.99
PANSS negative (baseline score)	−0.07	0.05	2.44	0.12	0.93	0.85	1.02
MADRS total (baseline score)	0.06	0.03	2.99	0.08	1.06	0.99	1.13
PAS total (baseline score)	−0.02	0.01	2.83	0.09	0.98	0.96	1.00
Verbal learning & memory	0.29	0.28	1.08	0.30	1.33	0.77	2.30
Severe impairment-improving trajectory *							
Intersection	−1.44	2.12	0.46	0.50			
Parental SES (medium-high)	0.81	0.61	1.76	0.18	2.25	0.68	7.46
PANSS positive (baseline score)	0.05	0.04	1.76	0.18	1.06	0.97	1.14
PANSS negative (baseline score)	−0.07	0.05	2.44	0.12	0.93	0.85	1.02
MADRS total (baseline score)	0.06	0.03	2.99	0.08	1.06	0.99	1.13
PAS total (baseline score)	−0.04	0.01	7.56	**<0.01**	0.96	0.93	0.99
Verbal learning & memory	1.13	0.42	7.25	**<0.01**	3.09	1.36	7.03

^a^ Nagelkerke R^2^ = 0.53, Model χ^2^ = 140.26, df = 24, *p* < 0.001. ^b^ Adjusted by age and sex. ^c^ Degrees of freedom: 1. * Reference category is *Severe impairment-Stable* trajectory. Abbreviations: SE: Standard Error; SES: Socioeconomic status; PANSS: Positive and Negative Syndrome Scale; MADRS: Montgomery–Åsberg Depression Scale; PAS: Premorbid Adjustment Scale. Bold type indicates *p* < 0.05.

**Figure A1:**
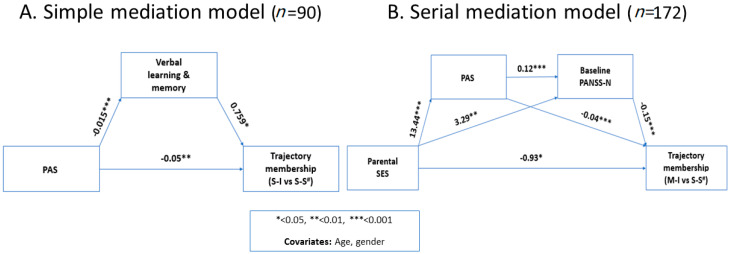
Mediation analyses. #: S-S was used as the reference category. Abbreviations: S-I: *Severe impairment-Improving;* S-S: *Severe impairment-Stable;* M-I: *Mild impairment-Improving.* PAS: Premorbid Adjustment Scale; SES: Socioeconomic Status, PANSS-N: Positive and Negative Syndrome Scale-Negative Subscale.

## Data Availability

The data presented in this study are available from the corresponding author on reasonable request.

## Supplementary Materials

The following are available online at https://www.mdpi.com/2077-0383/10/1/73/s1, Table S1: Comparison between participants who completed the assessment and those who dropped-out.

## Appendix A

Post-hoc mediation analyses

Given that previous works on FEP samples have suggested that premorbid adjustment may influence psychosocial functioning through verbal memory and negative symptoms [59], we decided to test how the identified predictors interact to impact functioning in our sample. For that, we examined mediation using a regression-based bootstrapping approach [60]. Analyses were performed with PROCESS [61]. Before beginning the analyses, two dummy variables for trajectory membership were created, one including only *Mild impairment-Improving* and *Severe impairment-Stable* trajectories and another including only *Severe impairment-Improving* and *Severe impairment-Stable* trajectories.

First: we used PROCESS model 4 to test a simple mediation model with trajectory membership (*Severe impairment-Improving* vs. *Severe impairment-Stable*, with *Severe impairment-Stable* trajectory as the reference category) as the outcome variable (Y), baseline PAS score as the predictor variable (X) and baseline verbal learning and memory as the mediator variable (M) (Figure A1). Age and sex were included as covariates. The data are consistent with the claim that better premorbid adjustment positively impacts verbal learning and memory, which in turn increases the probability to belong to the severe and improving functional impairment trajectory (indirect effect = −0.011; 95% CI = −0.030 to −0.001). The mediation partially explains the effect of premorbid adjustment on trajectory membership; in addition, premorbid adjustment influences class membership independently from the proposed mechanism (*b* = −0.05, *p* = 0.002). Hence, we infer complementary partial mediation [62].

Second: we used a series mediation model to assess mediation between predictors of *Mild impairment-Improving* vs. *Severe impairment-Stable* trajectory. In this model, trajectory membership (*Mild impairment-Improving* vs. *Severe impairment-Stable*, with *Severe impairment-Stable* trajectory as the reference category) was the outcome variable (Y) and parental SES the predictor variable (X). Baseline PAS score (M1) and baseline PANSS negative subscale scores (M2) were included, in this order, as mediator variables. Total MADRS score was not considered as a mediator as no association with parental SES was found in a preliminary analysis. We could establish a serial mediation from parental SES through premorbid adjustment and through baseline negative symptoms to trajectory membership (indirect effect = −0.249; 95% CI: −0.551 to −0.086). In addition, parental SES had an indirect effect on class membership only through premorbid adjustment (indirect effect = −0.525, 95% CI: −1.097 to −0.171) and only through baseline negative symptoms (indirect effect = −0.504, 95% CI: −1.076 to −0.135). Finally, there was a direct effect of parental SES on trajectory membership (*b* = −0.932, *p* = 0.029), indicating complementary partial mediation.
