# Supplementary material for: Exploring Risk and Resilient Profiles for Functional Impairment and Baseline Predictors in a 2-Year Follow-Up First-Episode Psychosis Cohort Using Latent Class Growth Analysis
Source: J Clin Med. 2020 Dec 28;10(1):73. doi: 10.3390/jcm10010073 (PMC7796026; doi:10.3390/jcm10010073)
Supplement: Supplementary file 1 [file jcm-10-00073-s001.pdf]

Table S1. Comparison between participants who completed the assessment and those who dropped-out.

	**Baseline**				**12-month follow-up**				**24-month follow-up**			
**BASELINE CHARACTERISTICS**	**Non-drop-out** ***n*** **= 261**	**Drop-out** ***n*** **= 14**	**U/X²**	***p***	**Non-drop-out** ***n*** **= 203**	**Drop-out** ***n*** **= 72**	**U/X²**	***p***	**Non-drop-out** ***n*** **= 157**	**Drop-out** ***n*** **= 118**	**U/X²**	***p***
**Age** (years) [a]	25.0(9)	24.7(10)	1,850	0.94	24.8(8)	25.4(10)	8,115.5	0.16	25.0(9)	25.1(8)	9903.5	0.33
**Sex** (Female) [b]	87(33.3)	6(42.9)	0.54	0.46	62(30.5)	31(43.1)	3.72	0.05	53(33.8)	40(33.9)	0.001	0.98
**Marital status** (Single) [b]	222(85.1)	11(78.6)	0.43	0.51	177(87.2)	56(77.8)	3.64	0.06	138(87.9)	95(80.5)	2.84	0.09
**Ethnicity** (Caucasian) [b]	228(87.4)	8(57.1)	9.97	**0.002**	181(89.2)	55(76.4)	7.13	**0.01**	138(87.9)	98(83.0)	1.30	0.25
**Parental socioeconomic status** (Medium-high) [b]	141(54.6)	5(35.7)	0.36	0.55	111(54.9)	35(48.6)	0.15	0.70	89(57.0)	57(48.3)	1.15	0.28
**Living situation** (Living independently) [b]	55(21.1)	5(35.7)	1.67	0.20	42(20.7)	18(25.0)	0.58	0.45	34(21.7)	26(22.0)	0.01	0.94
**Educational level** (Higher education) [b]	115(44.2)	5(35.7)	0.39	0.53	85(42.1)	35(48.6)	0.92	0.34	71(45.5)	49(41.5)	0.43	0.51
**Occupational status** (Active *) [b]	136(52.1)	6(42.6)	0.45	0.50	105(51.7)	37(51.4)	0.002	0.96	83(52.9)	59(50.0)	0.22	0.64
**Somatic comorbidity** (Yes) [b]	73(28.0)	1(0.1)	3.28	0.19	60(29.6)	14(19.4)	2.79	0.25	47(29.9)	27(22.9)	1.74	0.42
**Family history psychiatric disorder** (Yes) [b]	146(55.9)	4(28.6)	4.01	**0.04**	119(58.6)	31(43.0)	5.19	**0.02**	96(61.1)	54(45.8)	6.43	**0.01**
**Substance use** (Yes) [b]	47(18.0)	3(21.4)	40.97	**<0.001**	33(16.3)	17(23.6)	8.14	**0.02**	25(15.9)	25(21.2)	4.20	0.12

[a] Values are indicated as median (Interquartile Range). [b] Values are indicated as *n* (%). * Active includes workers and students. Bold type indicates $p < 0.05$.
